# The alarming consequences of workforce reductions at the FDA, EPA, NIH and CDC in the United States

**DOI:** 10.1007/s00204-025-04065-5

**Published:** 2025-05-13

**Authors:** Daniel R. Dietrich, Sonja von Aulock, Anna Bal-Price, Michael Coleman, Mark Cronin, Paul Jennings, Angela Mally, Mathieu Vinken, Matthew Wright, Martin van den Berg

**Affiliations:** 1 Human and Environmental Toxicology, University of Konstanz, Constance, Germany; 2 Alternatives to Animal Experimentation, Konstanz, Germany; 3 European Commission Joint Research Centre Ispra, Ispra, Italy; 4 Aston University, College of Health and Life Sciences, Birmingham, UK; 5 Liverpool John Moores University, School of Pharmacy and Biomolecular Sciences, Liverpool, UK; 6 Division of Molecular and Computational Toxicology, Vrije Universiteit Amsterdam, Amsterdam, Netherlands; 7 Department of Toxicology, Julius Maximilians University Würzburg, Würzburg, Germany; 8 Vrije Universiteit Brussel, Brussels, Belgium; 9 Newcastle University, Translational and Clinical Research Institute, Newcastle Upon Tyne, UK; 10 Utrecht University, Institute for Risk Assessment Sciences, Utrecht, Netherlands

We, the Editors-in-Chief of toxicology journals in Europe, are deeply troubled by the recent and proposed layoffs of regulatory scientists at the U.S. Center for Disease Control (CDC), National Institutes of Health (NIH), Food and Drug Administration (FDA) and the Environmental Protection Agency (EPA) under the Trump administration. This situation has raised our serious concerns regarding the future of public health, environmental protection, and scientific progress in the United States. As we examine the implications of these recent workforce reductions, it becomes increasingly evident that the consequences go far beyond the immediate loss of scientific personnel. In fact, they pose a threat to the very foundation of regulatory science and the safeguarding of public welfare and environmental health and thus will impact negatively on the safety of food, water, air, and medicines for every American.

Indeed, at the FDA, the budget cuts will significantly affect vital areas such as food safety, the regulation of drugs and medical devices, and the supervision of emerging technologies, including artificial intelligence in healthcare. Undoubtedly, these reductions will result in considerable delays in regulatory decisions, leading to a more cautious approach to the approval of new medical devices and pharmaceuticals. This caution, while possibly meaning to avoid harm, will slow or hinder innovation and postpone access to life-saving treatments for patients who urgently require them.

The circumstances at the EPA are equally alarming. The agency’s capacity to oversee and enforce environmental regulations has been significantly weakened because of these (proposed) workforce reductions. This reduction will undoubtedly impede the agency’s capability to ensure core responsibilities for the key basics of everyday life, i.e., clean water and air. Moreover, it will prevent the agency’s ability to tackle urgent environmental challenges, including climate change and environmental pollution, which scientists globally increasingly identify as existential threats to public health and the environment. Furthermore, the EPA’s role in regulating pesticides is vital for ensuring agricultural safety, thus the continued availability of healthy and sufficient foods, and safeguarding ecosystems. Prolonged delays in evaluating and approving both old and new pesticides create considerable uncertainty for agricultural sectors and may lead to significant economic repercussions. Such compromised oversight of existing and new pesticides could also have grave implications for public health. As toxicologists, we are concerned that these delays in evaluating new substances, reduced scrutiny of current chemicals, and weakened enforcement of safety standards are now becoming the standard practice rather than the exception in the United States.

In addition, the current loss of seasoned toxicologists at the government level may also significantly impede the development of innovative methodologies for toxicological testing and risk assessment of chemicals. In this respect, the quest for alternatives to animal testing and predictive models for chemical exposure is essential for enhancing our current safety protocols. However, with a reduced government workforce possessing toxicological expertise and experience, advancements in these crucial areas may stagnate, leaving consumers exposed to potentially unsafe products as well as environmental threats.

The wider ramifications of the cuts made and proposed by the Trump administration do not solely impact the domestic landscape in the United States but also extend into the international arena of public and environmental health. The United States’ exit from the World Health Organization (WHO) under the Trump administration further intensifies this predicament. Traditionally, U.S. contributions have represented about 20% of the WHO’s financial resources, facilitating essential initiatives in disease monitoring, pandemic readiness, and combating infectious illnesses. This withdrawal not only threatens these vital WHO programs but also raises alarms regarding the United States’ capacity to address emerging global health emergencies. By distancing itself from essential global health coalitions, the United States risks diminishing its authority and sway in global health diplomacy, leading to long-lasting consequences for public health and safety. In the absence of this support to the WHO, critical care initiatives may face interruptions, putting at risk vulnerable populations around the globe, including the USA, and particularly in low-income countries. As a consequence, this will undoubtedly lead to millions of preventable deaths across the world for which the current U.S. Government must be held accountable.

The recent restrictions imposed on the communications of NIH, CDC, FDA, and EPA scientists over the past few months have significant implications for scientific collaboration and transparency. By restricting government scientists to express their opinions and share research results at conferences, as well as to engage in peer reviews, the Trump administration inhibited the critical review, exchange, and spread of essential scientific discoveries. This disruption not only obstructs the peer-review process that is vital for upholding the quality and integrity of scientific inquiry but also raises serious concerns about the politicization of biomedical sciences. Such limitations must be seen as attempts by the Trump administration to control specific science-related policies or outcomes. These actions and specific communications from the current U.S. Government will further diminish public confidence in scientific institutions and their guidance provided on issues critical to health and well-being.

The interrelatedness of these issues cannot be emphasized enough. The restrictions and layoffs of FDA and EPA scientists, and just recently at NIH and CDC, have extensive repercussions, impacting not just the U.S. domestically, but also hindering international efforts in public health and environmental safeguarding. Historically, the U.S. has been a prominent leader in establishing health and environmental benchmarks. However, the latest political maneuvers undermine that credibility and will create a void that hopefully other nations with robust independent scientific expertise and economies, e.g., the EU, Canada, China, and Japan, are willing to address. Irrespective of the latter, the scientific knowledge and insights of U.S. Government scientists lost will not only be profoundly missed in the years ahead but will throw public health and safety back to the stone age of safety science.

As we gaze into the future, the timeline for the FDA, EPA, NIH, and CDC to recover from these restrictions and layoffs remains worrisome. Rebuilding their scientific workforce and regaining lost expertise will necessitate a sustained effort and dedication from future U.S. administrations. For this, it will require future U.S. Governments that have a more realistic perspective on the significance of health and environmental sciences. The process of restoration will be complex, yet it remains essential for safeguarding public health and the environment for future generations. As European Editors-in-Chief of a wide array of toxicology journals, we must guard and support policies that emphasize scientific independence, integrity, transparency, and the recruitment of talented scientists to ensure that U.S. regulatory agencies can perform again their crucial functions in the future.

Undoubtedly, we emphasize that the present layoffs and restrictions on biomedical research enforced by the Trump administration resulted in a deeply concerning situation for public health and environmental safety. The consequences of these actions are already profound and extensive, jeopardizing the very foundations of regulatory science, the responsibility of toxicologists to protect public health and the environment, and finally jeopardizing the very fabric of everyday life of Americans and other populations. It is essential that U.S. politicians fully acknowledge the urgency of the current situation and take all necessary measures to restore the integrity and capacity of U.S. regulatory agencies as soon as possible. The health and safety of our society depend on it, first within the U.S. itself, but also beyond.

We firmly believe, as do most if not all researchers engaged in public health and environmental protection, that the actions implemented by the Trump administration have been misguided and that the administration does not genuinely, openly, and deliberately aim to endanger the well-being of millions of Americans for the sake of short-term political advantage. Therefore, we eagerly await the complete restoration of FDA, EPA, NIH, and CDC scientists and their functions in the very near future.

**Dr. Daniel Dietrich**

Editor-in-Chief, *Chemico-Biological Interactions, Computational Toxicology, and Journal of Toxicology and Regulatory Policy*

**Dr. Sonja von Aulock**

Editor-in-Chief, *ALTEX—Alternatives to Animal Experimentation*

**Dr. Anna Bal-Price**

Editor-in-Chief, *Reproductive Toxicology*

**Dr. Michael Coleman**

Editor-in-Chief, *Environmental Toxicology and Pharmacology*

**Dr. Mark Cronin**

Editor-in-Chief, *Computational Toxicology*

**Dr. Paul Jennings**

Editor-in-Chief, *Toxicology *in Vitro

**Dr. Angela Mally**

Editor-in-Chief, *Toxicology Letters*

**Dr. Mathieu Vinken**

Editor-in-Chief, *Toxicology and NAM Journal*

**Dr. Matthew Wright**

Editor-in-Chief, *Food and Chemical Toxicology*

**Dr. Martin van den Berg**

Editor-in-Chief, *Regulatory Toxicology & Pharmacology* and* Current Opinion in Toxicology*

